# Proposing TODD-graphene as a novel porous 2D carbon allotrope designed for superior lithium-ion battery efficiency

**DOI:** 10.1038/s41598-024-56312-x

**Published:** 2024-03-14

**Authors:** E. A. J. Santos, K. A. L. Lima, L. A. Ribeiro Junior

**Affiliations:** 1 Institute of Physics, University of Brasília, Brasília, 70910-900 Brazil; 2 Computational Materials Laboratory, LCCMat, Institute of Physics, University of Brasília, Brasília, 70910-900 Brazil

**Keywords:** TODD-graphene, 2D carbon allotrope, Electronic properties, Mechanical properties, Optical properties, Materials for devices, Nanoscale materials, Materials science

## Abstract

The category of 2D carbon allotropes has gained considerable interest due to its outstanding optoelectronic and mechanical characteristics, which are crucial for various device applications, including energy storage. This study uses density functional theory calculations, ab initio molecular dynamics (AIMD), and classical reactive molecular dynamics (MD) simulations to introduce TODD-Graphene, an innovative 2D planar carbon allotrope with a distinctive porous arrangement comprising 3-8-10-12 carbon rings. TODD-G exhibits intrinsic metallic properties with a low formation energy and stability in thermal and mechanical behavior. Calculations indicate a substantial theoretical capacity for adsorbing Li atoms, revealing a low average diffusion barrier of 0.83 eV. The metallic framework boasts excellent conductivity and positioning TODD-G as an active layer for superior lithium-ion battery efficiency. Charge carrier mobility calculations for electrons and holes in TODD-G surpass those of graphene. Classical reactive MD simulation results affirm its structural integrity, maintaining stability without bond reconstructions at 2200 K.

## Introduction

Since the discovery of graphene in 2004^1,2^, several optical, mechanical, electronic, and thermodynamic studies have been conducted on this material, revealing its unique underlying properties, most related to its atom-thick 2D honeycomb-like arrangement. From that year on, various new 2D carbon-based allotropes have been computationally proposed^3–11^. Some of them have been recently synthesized, such as the 2D biphenylene network^12^, the multilayer $\gamma$-graphyne^13^, and the monolayer fullerene network^14^. Nevertheless, due to the successful synthesis of these carbon allotropes, the search for new materials capable of revolutionizing flat electronics has intensified even further.

One of the current trends in the research of new 2D carbon allotropes is the search for structures that differ structurally from graphene, such as those with large pores^15–25^. This trend is because nanomaterials with distinct and broader ring structures demonstrate a superior capacity for the absorption of lithium atoms compared to graphene^26–32^. Examples of these structures with atomic thickness also include the pop-graphene^9^ with five, eight, and five-atom rings, 8-16-4-Graphyne^10^, Irida-graphene^11^ with five, eight, and six-atom rings, and 2D biphenylene network^12^ characterized by regularly arranged 4-, 6-, and 8-membered rings comprising sp^2^-hybridized carbon atoms. Drawing from the results regarding Li storage capacity, proposing additional porous 2D carbon-based materials featuring interconnected porous rings could offer new channels for energy storage applications.

In 2D materials, the band gap is often highly responsive to external stimuli such as tensile stress or compressive strain^33^. This tunability has been a critical feature in tailoring the electronic properties of various 2D materials for diverse applications^34–37^. In this context, the quest for unconventional 2D materials that can resist significant changes in their band structure under moderated stress regimes is actual. Such materials, capable of maintaining their optoelectronic properties despite external stress, hold considerable promise for developing robust and versatile components in flexible optoelectronics. Consequently, this study aims to contribute to exploring this possibility.

**Figure 1:**
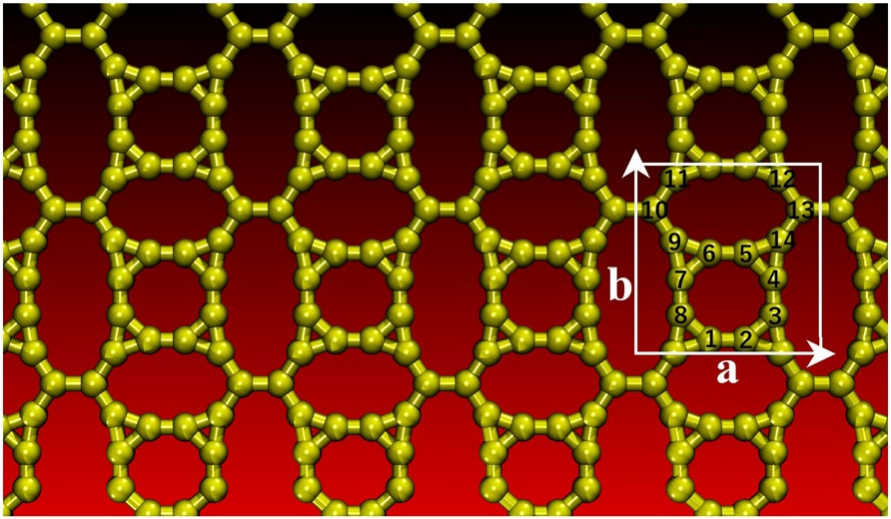
A diagram illustrating the lattice topology of TODD-G, where the unit cell is emphasized by a white rectangle defined by lattice vectors a and b. This figure was prepared using the VMD software^39^.

In this work, we used a computational protocol to propose an innovative 2D carbon allotrope characterized by 3-8-10-12-membered rings of sp^2^-hybridized carbon atoms, referred to as TODD-Graphene (TODD-G, see Fig. 1) from a bottom-up approach. We investigated its electronic, optical, and mechanical properties using density functional theory (DFT) and ab initio molecular dynamics (AIMD) simulations. Our findings show that TODD-G is metallic and structurally stable, as indicated by its integrity at 1800 K through classical reactive MD simulations (ReaxFF). Its phonon dispersion signature without imaginary phonon modes further indicates its dynamic stability. This material exhibits optical activity across the visible and ultraviolet regions. TOOD-G has a low average diffusion barrier (about 0.85 eV) and a metallic framework boasting excellent conductivity, showing potential as a prospective anode material for lithium-ion batteries. Moreover, the results of the classical reactive MD simulations suggested that TODD-G maintains its structural integrity without any bond reconstructions at 1800 K.

## Results

### Structure and stability

We begin our analysis by presenting the structural characteristics of TODD-G. Geometry optimization calculations resulted in consistent lattice parameters, and both the PBE and HSE06 methods were employed. Here, we focus on presenting the results obtained within the HSE06 scheme. Figure 1 illustrates TODD-G’s atomic arrangement and unit cell with related lattice vectors. The unit cell has 14 atoms, with dimensions $a=7.03$ Å and $b=6.54$ Å. It exhibits an orthorhombic structure within the *P*1 space group. TODD-G forms a flat and periodic arrangement of sp^2^-hybridized carbon atoms featuring interconnected 3-8-10-12-membered rings. See Table 1 for a more comprehensive view of its bond configuration. Despite the unique topology of TODD-G, it is worth noting that the interatomic distances in this material align with those found in other 2D carbon allotropes^3,38^, underscoring its structural consistency.

It is worth noting that the planar density of TODD-G is 0.30 atom/Å^2^, surpassing that of graphyne (0.29 atom/Å^2^)^40^ and graphdiyne (0.23 atom/Å^2^)^41^. Yet, it falls short of graphene (0.38 atom/Å)^42^ and DHQ-graphene (0.33 atom/Å^2^)^43^. TODD-G exhibits a formation energy of $-8.25$ eV/atom, surpassing that of graphene ($-9.220$ eV/atom)^44^, indicating its lower stability than the latter.

**Table 1:** Distances between the highlighted atoms in Fig. 1 for bond analysis..

Bond type	Bond length (Å)	Bond type	Bond length (Å)
C1–C2	1.368	C9–C7	1.435
C2–C3	1.424	C9–C6	1.392
C3–C4	1.368	C9–C10	1.410
C4–C5	1.424	C10–C11	1.410
C5–C6	1.368	C12–C13	1.410
C6–C7	1.424	C13–C14	1.410
C7–C8	1.368	C5–C14	1.392
C1–C8	1.424	C4–C14	1.435

**Figure 2:**
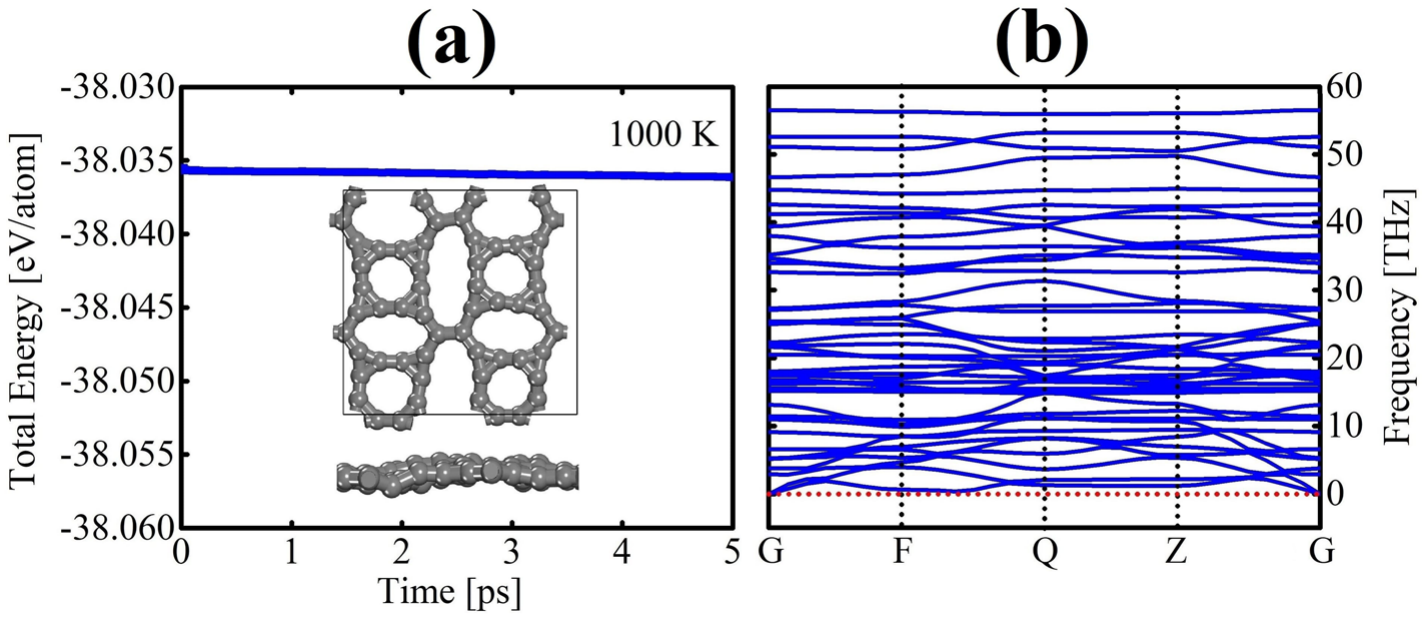
(**a**) The temporal progression of the total energy per atom lattice at 1000 K and (**b**) the phonon band structure of TODD-G, both computed using the PBE level. The insets in panel (**a**) depict top/side views of TODD-G at 5 ps.

To evaluate the thermal stability of TODD-G, we conducted AIMD simulations as illustrated in Fig. 2a. The Supplementary Material presents the video from where these AIMD snapshots were extracted. In these simulations, we monitored the temporal variations in the total energy per atom over a 5 ps duration, subjecting the material to a temperature of 1000 K (Supplementary Video 1). One can observe that variations in the total energy exhibit a nearly uniform pattern with minimal variations. The MD snapshots in Fig. 2a illustrate subtle deviations in TODD-G’s initial planarity and bond distances due to the increased temperature, with no occurrences of bond breaks or reconstructions. At 1000 K, the final configuration of TODD-G remains in line with the optimized structure (refer to Fig. 1).

For a more in-depth analysis of the dynamical stability of TODD-G, we calculated its phonon dispersion as illustrated in Fig. 2b. No imaginary frequencies are evident, signifying the intrinsic dynamical stability of TODD-G. The lack of a band gap between acoustic and optical modes indicates a scattering rate and relatively shorter phonon lifetimes, thereby influencing this material’s moderate lattice thermal conductivity. A well-established principle is that higher phonon frequencies correspond to stronger chemical bonds. In the case of TODD-G, the highest phonon frequency is approximately 56.50 THz, slightly surpassing the 49.11 THz observed in graphene^45,46^. The fused 3-8-atom rings in TODD-G have stiff bonds, forbidding atoms to oscillate more freely when contrasted to the graphene case.

**Figure 3:**
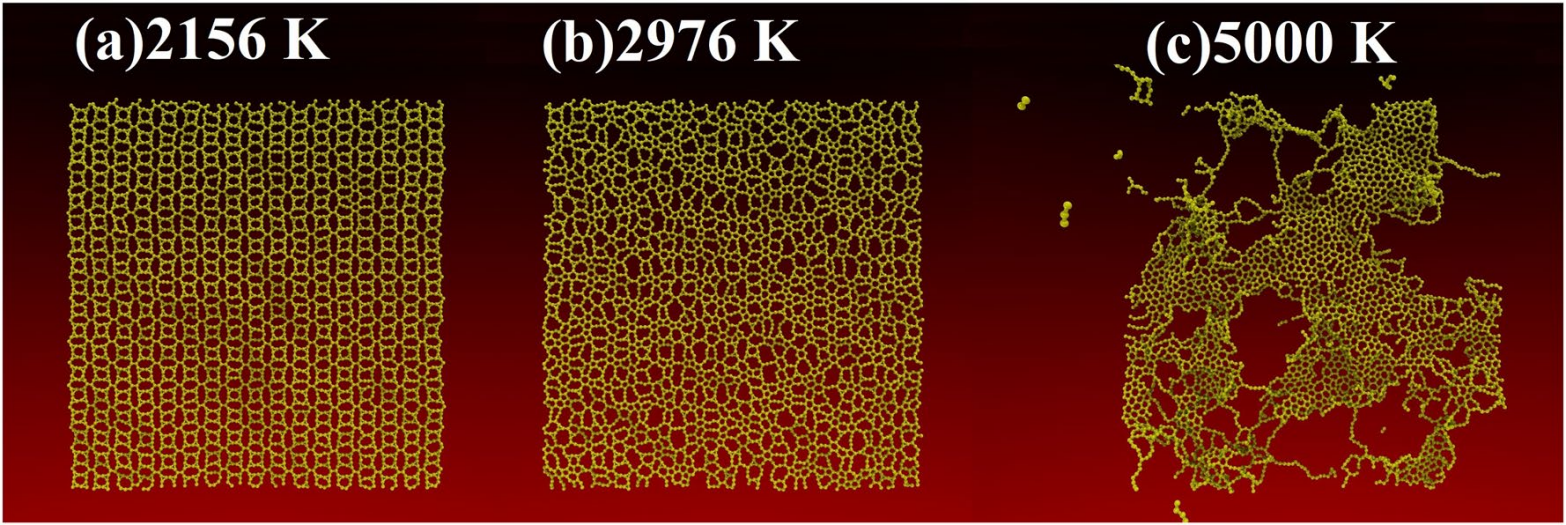
Classical reactive MD snapshots depicting the heating ramp process of TODD-G at various temperatures: 2156 K (**a**), 2976 K (**b**), and 5000 K (**c**), highlighting crucial temperature points for potential phase transitions in its topology. This figure was prepared using the VMD software^39^.

Figure 3 presents key classical reactive MD snapshots illustrating the dynamic response of the TODD-G monolayer during the heating simulation. For Initially, TODD-G was brought to thermal equilibrium at 300 K. This equilibrium was achieved while maintaining zero pressure in the *x* and *y* directions. The equilibrated structure is stable at 2156 K, as illustrated in Fig. 3a. Around 2200 K, TODD-G undergoes a phase transition, yielding a monolayer amorphous carbon (MAC) with a similar structure studied in references^47–49^, as depicted in Fig. 3b. This transition is evident in Fig. 3b, where the system exhibits the coexistence of linear atomic chains (LACs) and carbon rings formed with a distinct number of atoms. At 5000 K, the system has melted, giving rise to multiple clusters, including dispersed LACs and MAC domains scattered throughout the simulation box, illustrated in panel Fig. 3c.

**Figure 4:**
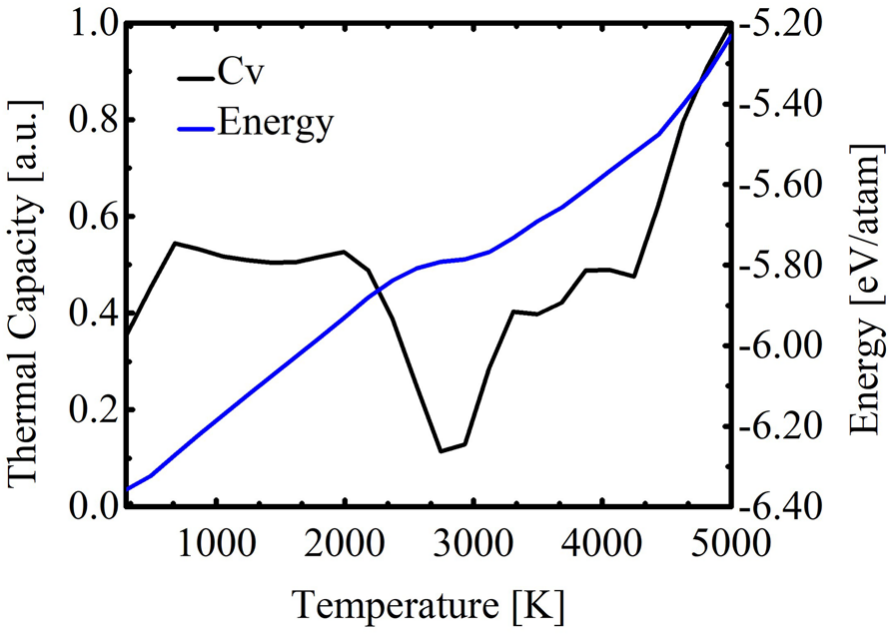
Total energy (blue) and heat capacity ($C_V$, black) values as a function of temperature for the TODD-G monolayer.

In Fig. 4, the blue curve represents the variation of total energy. In contrast, the black curve illustrates the heat capacity ($C_V$) as a function of temperature. The total energy exhibits a quasi-linear increase in the temperature range of 300–2400 K, followed by a quasi-parabolic behavior from 2500K-5000K. The most prominent peak in the $C_V$ curve corresponds to a melting point of approximately 2744 K (see Fig. 4). The TODD-G lattice remains intact during the initial heating stage (300–2600 K). However, at 2700 K, thermal vibrations induce structural changes, initiating the melting process during the second heating stage (2700–5000 K).

### Electronic and optical properties

**Figure 5:**
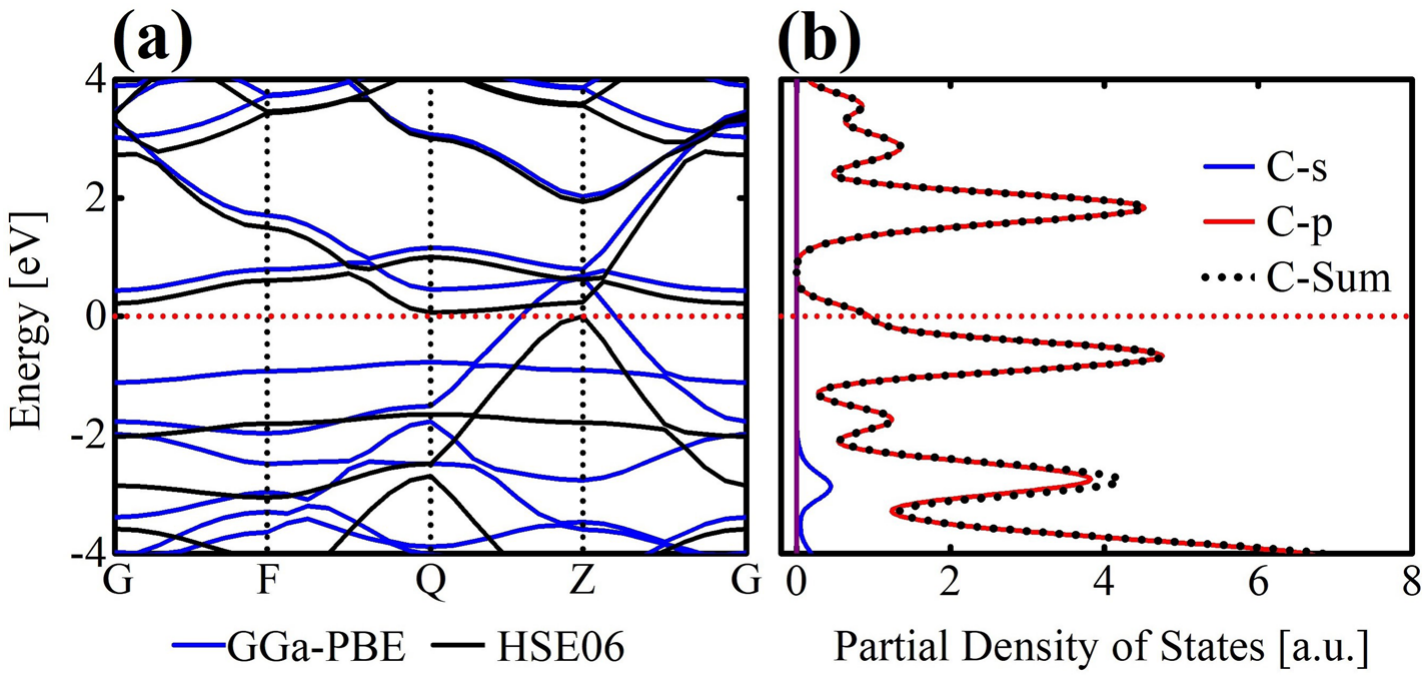
(**a**) Illustration of the electronic band structure, and (**b**) presentation of the partial density of states (PDOS) for TODD-G. The band structure was derived employing both the PBE (depicted in blue) and HSE06 (depicted in black) approaches, while PDOS calculations were conducted at the HSE06 level.

Next, we delve into the electronic properties of TODD-G.In Fig. 5a, the band structures are illustrated and computed using both the PBE and HSE06 methods. Figure 5b provides the PDOS exclusively for the HSE06 method. The band structure obtained with HSE06 showcases a small gap opening of approximately 0.06 meV. In contrast, the PBE-derived band structure does not reveal a gap opening. Both approaches point to a metallic signature for TODD-G. It is worth mentioning that PBE calculations often underestimate band gaps. In contrast, HSE06 calculations typically accurately describe a material’s electronic and optical properties. Figure 5a further highlights the intrinsic anisotropic conductance of TODD-G. Along the Q–Z direction, it exhibits metallic characteristics while manifesting semiconducting behavior along other paths.

Figure 5b depicts the PDOS for TODD-G. This figure reveals that p-states dominate, indicating their primary role in driving electronic transitions and interactions within TODD-G. Directional bonding phenomena are commonly associated with p-orbitals, in contrast to the minor contribution of s-states to the valence levels. In comparison, s-states make a minor contribution to the valence levels. The PDOS results unequivocally establish TODD-G as a metallic material.

Here, we highlight carrier mobility’s anisotropic behavior in the TODD-G. Specifically, the carrier mobility along the x-direction significantly exceeds that in the y-direction, a trend owing to the smaller effective mass of carriers in the x-direction. The Supplementary Material presents the electron and hole mobility calculation data. The calculated mobilities are 89.25/11.13 and 77.23/10.16 10^3^ cm^2^ V^-1^ s^-1^ for electrons/holes in x and y directions, respectively. TODD-G exhibits enhanced mobility compared to graphene, which can reach 30.0 10^3^ cm^2^ V^-1^ s^-1^^50^. Compared to graphene, the enhanced charge carrier mobility in TODD-G can be ascribed to their distinctive topologies.

**Figure 6:**
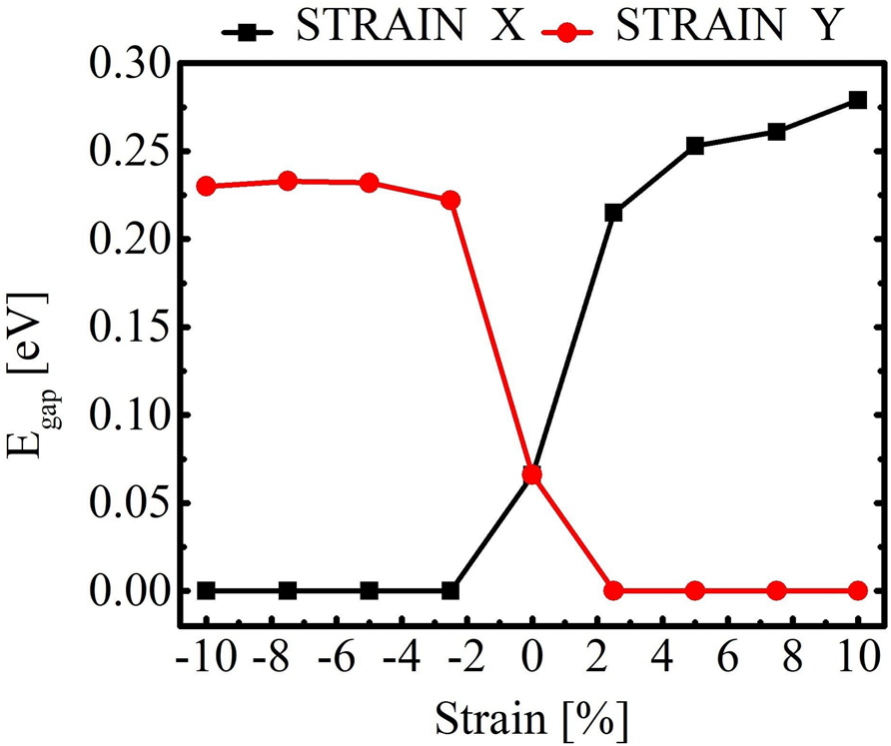
Gap-opening versus tensile/compressive stress in TOOD-G.

An unconventional trait was observed in TODD-G regarding its band structure as a response to applied strain. Figure 6 indicates that the band structure of TODD-G is not easily tunable by strain, as the gap-opening energy ($E_{gap}$) is relatively small, measuring about 0.27 eV for tensile stress in the x-direction and 0.22 eV for compressive strain in the y-direction.

Remarkably, compressive/tensile stress in the x-direction/y-direction results in a complete inversion in the gap-opening trend discussed above, effectively reducing it to zero. This unusual behavior is attributed to the unique topology of TODD-G, which introduces anisotropy in its electronic properties. It is distinct from other materials, like graphene^33^, where tensile and compressive stress might more uniformly impact the electronic properties considering directions parallel to the basal plane.

**Figure 7:**
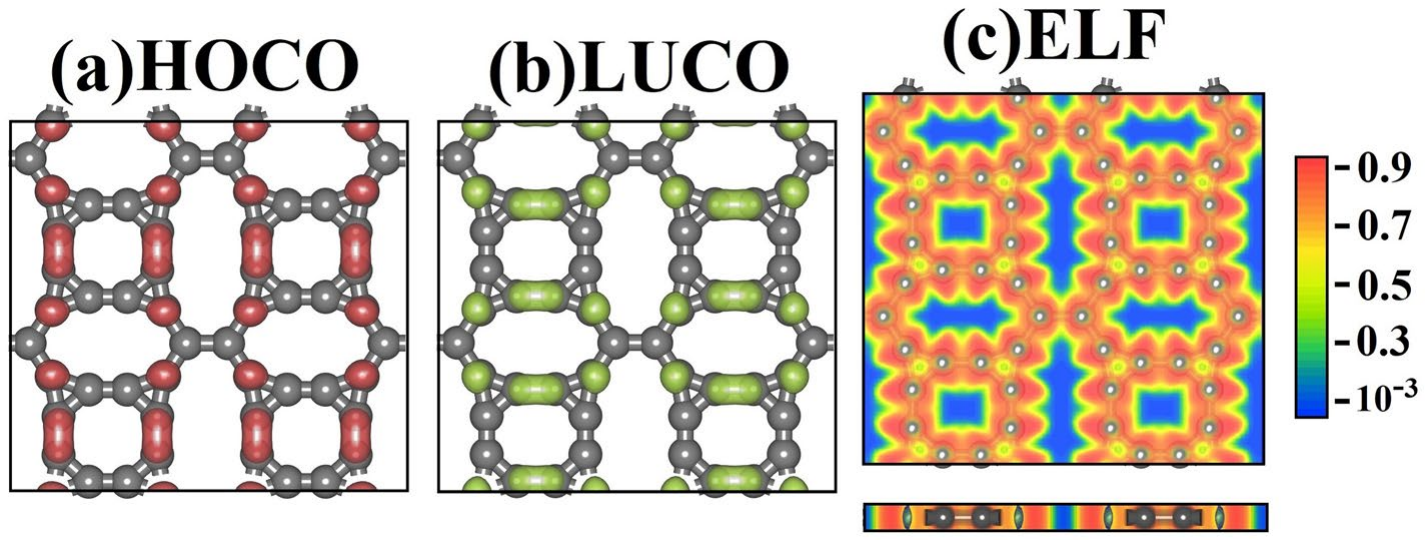
Panel (**a**) displays the highest occupied crystalline orbital (HOCO, highlighted in red), while panel (**b**) showcases the lowest unoccupied crystalline orbital (LUCO, presented in green). In Panel (**c**), the electron localization function (ELF) is schematically depicted. This figure was prepared using the Materials Studio Visualizer^51^.

For a deeper understanding of the chemical interactions within TODD-G, Fig. 7 shows the highest occupied crystalline orbital (HOCO), lowest unoccupied crystalline orbital (LUCO), and the electron localization function (ELF). Figure 7a,b illustrate the localization of HOCO and LUCO in TODD-G, respectively. The HOCO primarily localizes on the 8-membered ring, indicating a higher concentration of low-energy electrons or a higher electron-charge density in this region. LUCO mainly resides on the bonds of the decagons, suggesting a lower density of low-energy electrons or the availability of lower-energy electrons for engaging in chemical interactions in this particular region. This orbital distribution results in a charge imbalance, with the octagonal rings having most of the negative charge.

In addition to examining the orbital localization in TODD-G, Fig. 7c showcases the Electron Localization Function (ELF). ELF helps to identify regions with electron localization/delocalization. Values nearing 1.0 signify strong covalent interactions or the presence of lone pair electrons, and values lower than 0.5 point to electron delocalization, ionic bonds, or weak Van der Waals interactions.

The color map for ELF in TOOD-G shows a significant electron concentration around the octagonal ring, with values ranging from 0.7 to 1.0. In contrast, bonds between C–C atoms in other lattice regions are characterized by lighter-yellow areas with values near 0.5, indicating a degree of electron delocalization. Materials with valence electrons exhibiting delocalization display metallic-like conductivity, allowing for free-like electron transport. Conversely, materials featuring strong covalent bonds often exhibit semiconductor-like conductivity. In the case of TODD-G, the coexistence of localized and delocalized electrons within its lattice underlies the anisotropic conductance observed in its electronic band structure, as discussed earlier. This complex electron distribution pattern contributes to its unique electronic properties and conductivity behavior.

**Figure 8:**
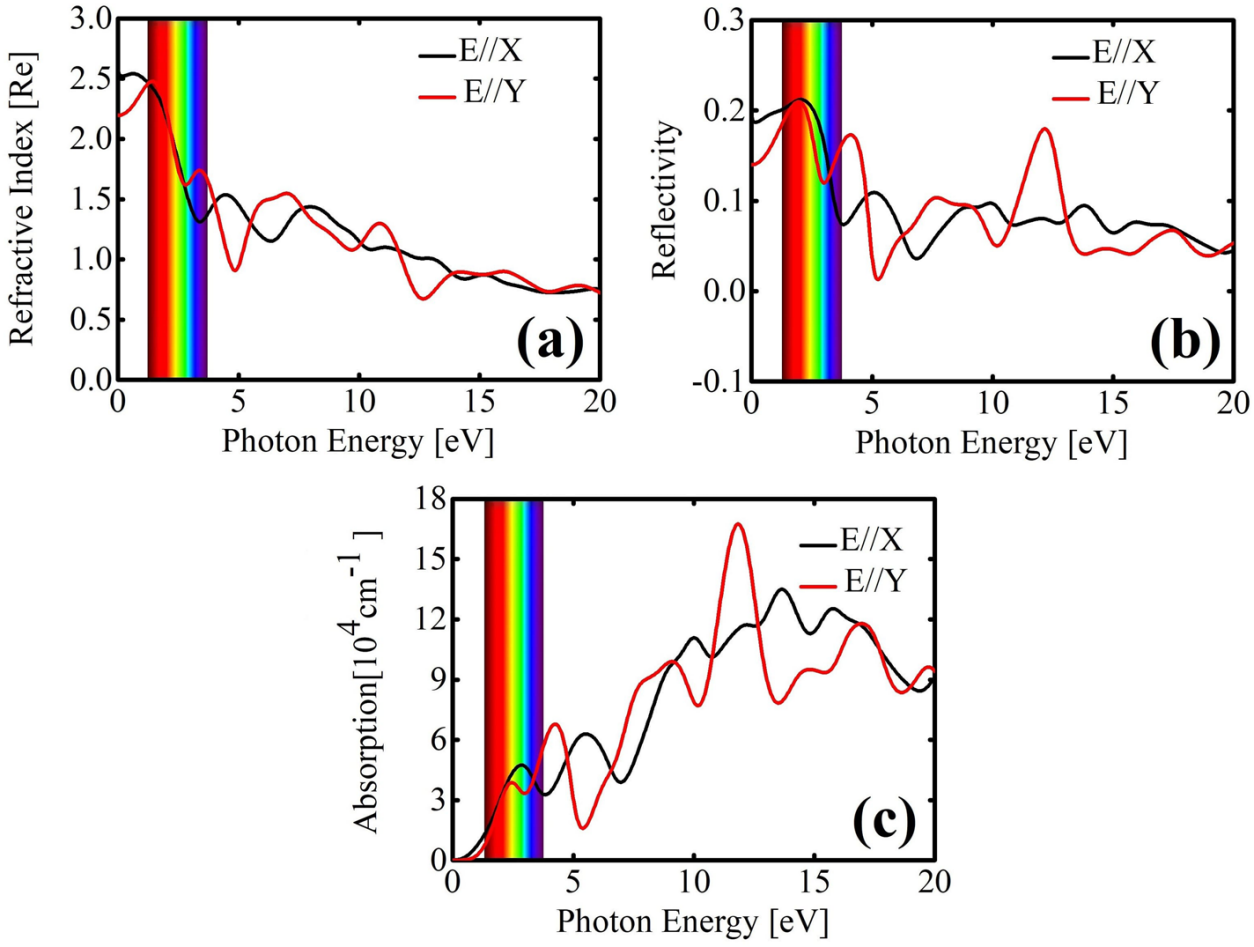
Interplay between the optical properties and the photon energy for TODD-G.

Materials exhibit electronic transitions encompassing interband and intraband processes, crucial determinants of their optical behavior. The lattice topology of TODD-G introduces anisotropy in the underlying optoelectronic properties, endowing distinct optical features along the in-plane polarization directions. In Fig. 8, we delve into the optical characteristics of TODD-G, polarizing the light along the *x* and *y* directions, denoted as E//X and E//Y, respectively. Using the HSE06 method for calculations, we explore optical parameters refractive index, reflectivity, and the absorption coefficient, all derivable from the complex dielectric function^52^.

Birefringence occurs when the speed of light varies in different polarization directions in the material. In TODD-G, the refractive indices along polarization directions parallel to its basal plane exhibit anisotropy, as depicted in Fig. 8a. The most significant refraction occurs at the infrared limit. This observed trend indicates that TODD-G possesses birefringent properties, setting it apart from graphene^53^. Moreover, graphene has an intense refraction activity within the Vis-UV ranges^53^. There is a decline in the refractive index value, converging to 0.07 for values above 18 eV for the photon energy. This convergence indicates that incident UV light is refracted similarly in all directions. The reflection function quantifies the proportion of photon energy reflected from a surface compared to the incident photon energy. In Fig. 8b, the TODD-G reflectivity coefficient is presented as a varying photon energy function in the *x* and *y* directions. Across a range of photon energies from 0 to 20 eV, the reflectivity coefficients remain below 0.03. The maximum reflectivity occurs within the visible region, suggesting efficient transmission of incident light with minimal impact on TODD-G. Reflectivity peaks are observed for photon energies within the range of 10–15 eV, with the reflectivity that reaches its peak at 1.65 eV, attaining a maximum reflectivity value of 0.026. This analysis underscores TODD-G’s sensitivity in the reflection of incident light within the Vis-UV spectrum, akin to findings in graphene^53^. Reflectivity peaks that decrease with increasing energy may indicate the existence of electronic resonances or specific vibrations in the material corresponding to these energies. These observations suggest that incident light on TODD-G is primarily absorbed, indicating its lack of transparency.

Figure 8c displays the optical absorption coefficient spectra, outlining how light strength diminishes per unit distance through the material. As seen in this figure, TODD-G exhibits a high absorption coefficient (10^4^ cm^-1^), reflecting its metallic properties. The first absorption peaks for E//X and E//Y fall within the visible spectrum, a notable departure from observations in graphene^53^. Specifically, TODD-G’s initial peak, at approximately 2.9 eV (located in the red region of the visible spectrum), shows a red-shift of about 1.1 eV compared to graphene, which has its first peak in the UV region (around 4.0 eV)^53^. These optical findings suggest that TODD-G holds promise for applications as detectors and absorbers in the Vis-UV ranges of the spectrum.

### Mechanical properties

**Figure 9:**
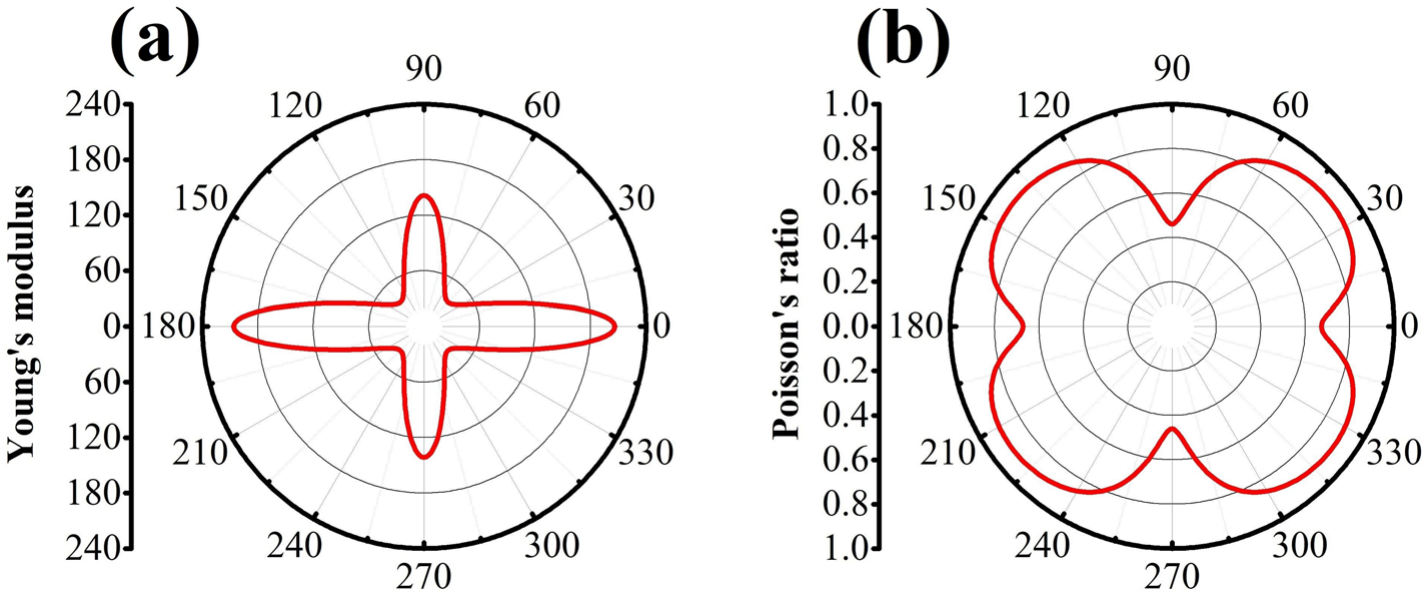
(**a**) Poisson’s ratio and (**b**) Young’s modulus for the basal plane of TODD-G.

We proceed to examine the elastic properties of TODD-G. In exploring the material’s mechanical anisotropy, we evaluate the Poisson’s ratio ($\nu (\theta )$) and Young’s modulus ($Y(\theta )$) under pressure in the xy plane^54,55^.

1 $$\begin{aligned} \displaystyle Y(\theta ) = \frac{{C_{11}C_{22} - C_{12}^2}}{{C_{11} \alpha ^4 + C_{22}\beta ^4 + \left( \frac{{C_{11}C_{22} -C_{12}^2}}{{C_{44}}} - 2C_{12}\right) \alpha ^2\beta ^2}} \end{aligned}$$

and

2 $$\begin{aligned} \displaystyle \nu (\theta )= \frac{{ \left( C_{11} + C_{22} -\frac{{C_{11}C_{22} - C_{12}^2}}{{C_{44}}} \right) \alpha ^2\beta ^2 -C_{12} \left( \alpha ^4 + \beta ^4 \right) }}{{C_{11}\alpha ^4 + C_{22}\beta ^4 +\left( \frac{{C_{11}C_{22} - C_{12}^2}}{{C_{44}}} -2C_{12}\right) \alpha ^2\beta ^2}}, \end{aligned}$$

where $\alpha =\cos (\theta )$ and $\beta =\sin (\theta )$. The elastic constants of TODD-G are shown in Table 2. Figure 9 depicts a 2D representation of Young’s modulus and Poisson’s ratio (Fig. 9a,b) in the xy plane for this material.

**Table 2:** A summary for the C_ij_ (GPa) values, maximum value for Young’s modulus (GPa) ($Y_{MAX}$), and maximum ($\nu _{MAX}$) and ($\nu _{MIN}$) Poisson’s ratios..

Structure	C_11_	C_12_	C_22_	C_44_	$Y_{MAX}$	$\nu _{MAX}$	$\nu _{MIN}$
TODD-G	204.57	137.47	298.66	9.61	106.20	0.65	0.30

The elastic constants C11, C22, C12, and C44, are detailed in Table 2, meet the Born-Huang criteria for an orthorhombic crystal ($C_{11}C_{22} - C_{12}^2>0$ and $C_{44}>0$)^56,57^, affirming its robust mechanical stability. Furthermore, we computed Young’s modulus and Poisson’s ratio for TODD-G using Eqs. 1 and 2 (refer to Fig. 9). These properties exhibit distinct anisotropic characteristics.

As expected, TODD-G displays anisotropic behavior in Young’s modulus values when it undergoes deformation due to its distinctive ring arrangement within its plane (refer to Fig. 9a). The corresponding values $Y_{MAX}$ for the deformation in the x and y directions are approximately 121 GPa and 195 GPa, respectively, nearly a tenth of the value reported for graphene (1.0 TPa^58^). This difference can be attributed to the intrinsic porosity in TODD-G, which arises from the presence of 8-10-12-atom rings and the bond stiffness within fused trigonal rings.

Common materials typically exhibit a Poisson ratio ranging from 0.2 to 0.5^59^. A Poisson ratio 0.5 denotes incompressible materials, indicating minimal change in their lateral dimensions under strain. When subjected to uniaxial tensile loading in the x direction, TODD-G displays a maximum Poisson ratio ($\nu _{MAX}$) 0.65. This value exceeds that observed in graphene, which is approximately 0.19^60^. The heightened Poisson ratio of TODD-G can be attributed to its lattice arrangement, characterized by a higher porosity than that of graphene. This increased porosity enables TODD-G to undergo more deformation under tension than graphene, resulting in an elevated Poisson ratio. The intrinsic anisotropy of TODD-G is also evident in Fig. 9b, where the minimum Poisson’s ratio ($\nu _{MIN}$) of about 0.30 occurs under strains applied in the y-direction, indicating TODD-G’s relative incompressibility in this scenario.

### Lithium-ion adsorption on TODD-G

**Figure 10:**
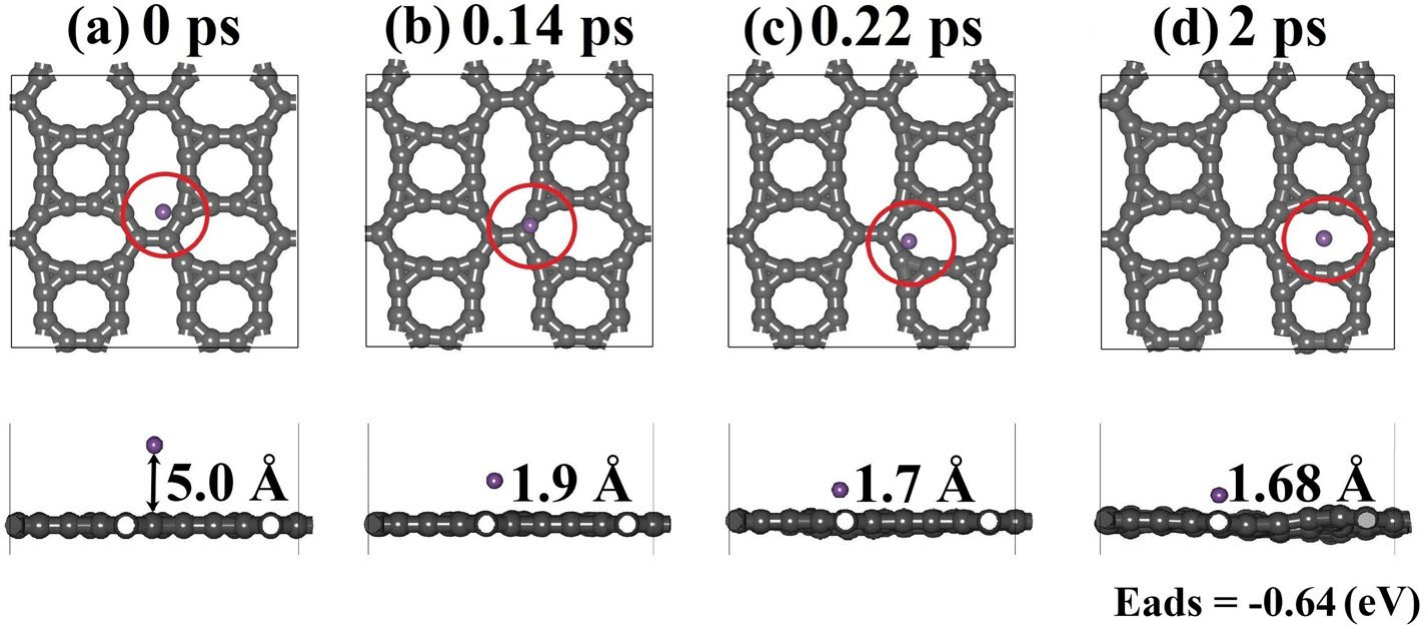
AIMD snapshots for Li adatom diffusion on TODD-G at 500K. This AIMD simulation includes vdW corrections. This figure was prepared using the Materials Studio Visualizer^51^.

Since Li storage is a primary application focus for 2D carbon-based materials^61,62^, we now delve into the Li adsorption process on TODD-G. AIMD simulations, with van der Waals (vdW) corrections within the Grimme framework^63,64^, were conducted to investigate the dynamical stability of a system comprising TODD-G and a single Li adatom (see Fig. 10). The Supplementary Material presents the video from where these MD snapshots were extracted. The initial TODD-G/Li system for these simulations was generated using the uncoupled Monte Carlo (UMC) approach facilitated by the Adsorption Locator Modulus of Materials Studio^65,66^. It is worth mentioning that voltage profile analysis can also be used to evaluate the material performance in Li-ion battery applications^67–72^.

The UMC approach involves sampling various trial arrangements in the canonical ensemble by creating an extensive set of TODD-G/Li systems, beginning with a trial configuration. These conformations were randomly selected by translating the Li adatom parallel and perpendicular to the TODD-G plane. The quest for the structures with the lowest energy levels is facilitated through the simulated annealing method, with the Metropolis algorithm providing the statistical weights for this process^66^. Further details on the UMC approach used in this study can be found in reference^73^.

In Fig. 10a, the AIMD snapshot depicts the initial system at 0 ps. UMC calculations yield a system with the lowest energy, positioning the Li adatom 5.0 Å above the TODD-G surface. Consistent with previous studies, lithium atoms prefer absorbing on the hollow sites of 2D carbon allotropes^9,30,74^. This pattern is also observed in TODD-G, as illustrated by AIMD snapshots in Figs. 10b–d, showcasing the rapid migration of the Li adatom from the 12-atom ring towards the 10-atom ring within 2 ps. The ultimate adsorption energy of Li and the corresponding distance are approximately $-0.64$ eV and 1.68 Å, respectively. Li displays high mobility on TODD-G and interacts weakly with its surface. Importantly, this adsorption energy is comparable to the values reported for other theoretically predicted 2D carbon allotropes popgraphene ($-0.57$ to $-0.95$ eV^9^), net-$\tau$ ($-0.37$ to $-0.60$ eV^29^), and C_5678_ ($-0.42$ to $-0.52$ eV^30^).

These AIMD snapshots highlight that, in TODD-G, the most favorable position for Li interaction is the porous region defined by the 10-atom ring, emphasizing a preference for hollow sites over bridge sites. The adsorption energy $E_{ads}=(E_{system}-E_{TODD-G}-nE_{Li})$, where $E_{system}$ and $E_{TODD-G}$ represent the total energies of the TODD-G structure after and before Li adsorption, and $E_{Li}$ denotes the energy/atom in the 3D phase of metallic Li.

**Figure 11:**
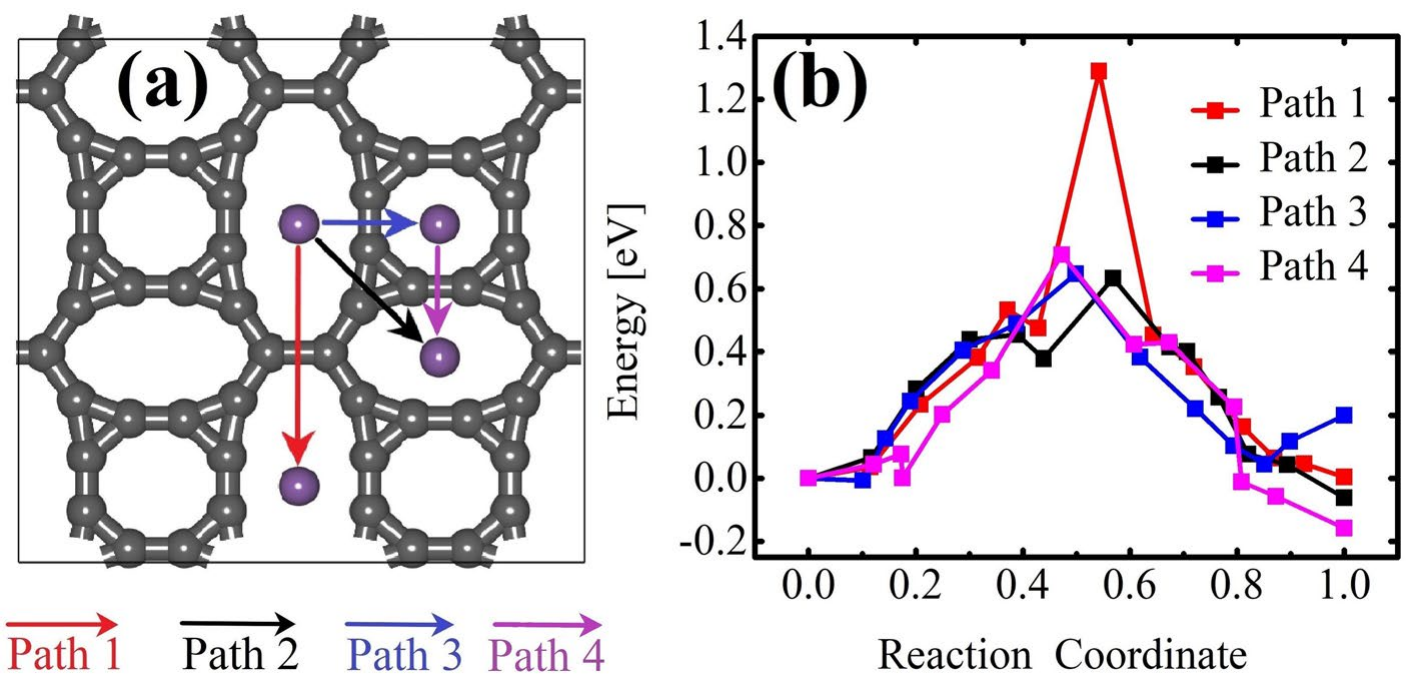
Three potential migration routes (**a**) and their associated energy profiles (**b**) for Li diffusion on a TODD-G sheet. These simulations incorporate van der Waals (vdW) corrections.

We selected three potential migration pathways along distinct directions to explore Li mobility in TODD-G and calculated their related barrier. The transition states for these migration pathways are depicted in Fig. 11a. The pathways considered are as follows: Pathway 1 from the 12-atom ring to another 12-atom ring (red line), Pathway 2 from the 12-atom ring to the 10-atom ring (black line), Pathway 3 from the 12-atom ring to the 8-atom ring (blue line), and Pathway 4 from the 8-atom ring to the 10-atom ring (pink line).

In examining these pathways, it was observed that the barrier between neighboring 12-atom rings (Path 1) is higher, measuring approximately 1.28 eV (see Fig. 11b). The diffusion barriers along Paths 2, 3, and 4 are 0.63 eV, 0.64 eV, and 0.71 eV, surpassing that of graphene (0.31 eV)^75^, as shown in Fig. 11. Although the maximum diffusion barrier of TODD-G is overcome by those for graphene and $\Psi$-graphene (approximately 0.31 eV)^28^, it remains comparable to those of $\Theta$-graphene (0.48 eV)^76^, xgraphene (0.49 eV)^77^, popgraphene (0.55 eV)^9^ and C_5678_ (0.44 eV)^30^. It is worth mentioning that it is considerably lower than the biphenylene network (2.44 eV) and phagraphene (2.07 eV)^74^. Li atoms on TODD-G have an average migration barrier of 0.85 eV. The minimal barrier implies favorable Li-ion mobility, indicating a promising charge/discharge rate for TODD-G, which can be crucial for lithium-ion battery applications.

**Figure 12:**
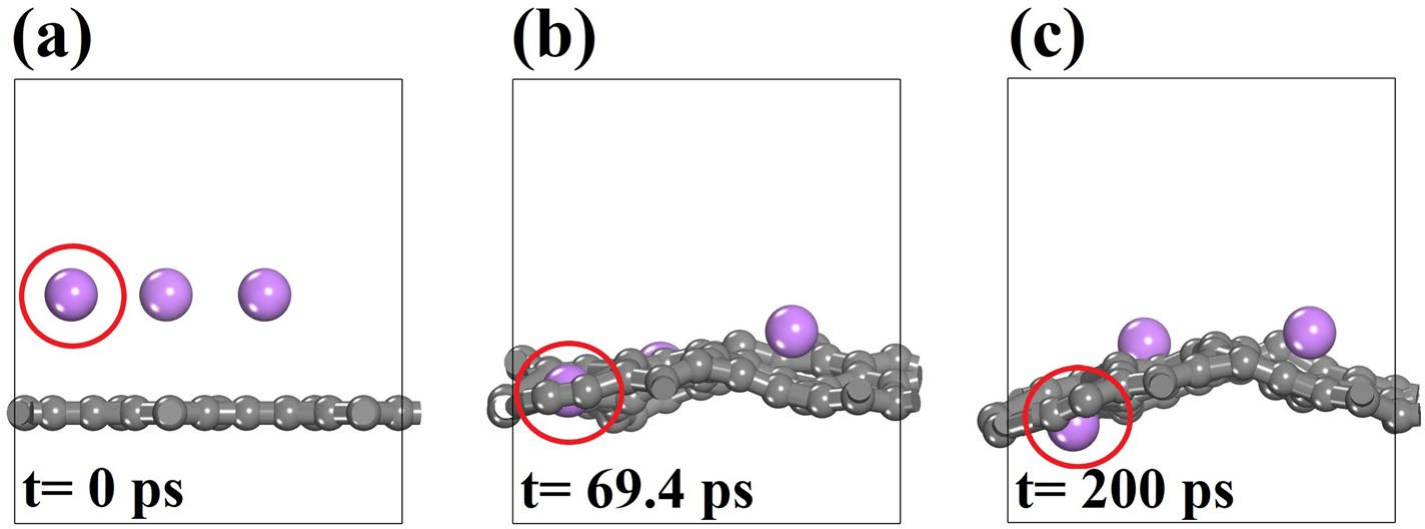
AIMD snapshots for three Li adatom diffusion on TODD-G at 500 K. This AIMD simulation includes vdW corrections. The Li atom that passes through the TODD-G surface is highlighted with a red circle. This figure was prepared using the Materials Studio Visualizer^51^.

At room temperature, Li atoms predominantly adsorb on TODD-G within the 10- and 12-membered rings. The energy barrier for Li transition across the material plane in these scenarios ranges from 1.35 to 1.45 eV. It is worth noting that this range of energy barriers is higher than that for Li diffusion on the TODD-G surface (see Fig. 12). However, with an increased Li concentration (at least for three atoms), the TODD-G lattice undergoes distortions, reducing the energy barrier for a Li atom to pass through the 10- and 12-membered rings, as depicted in Fig. 11. This figure shows representative AIMD snapshots for the dynamics of three Li atoms adsorbed on the TODD-G surface. The distortions caused by several Li adsorptions tend to increase the ring’s diameter, enabling the diffusion of these ions through the material plane.

**Figure 13:**
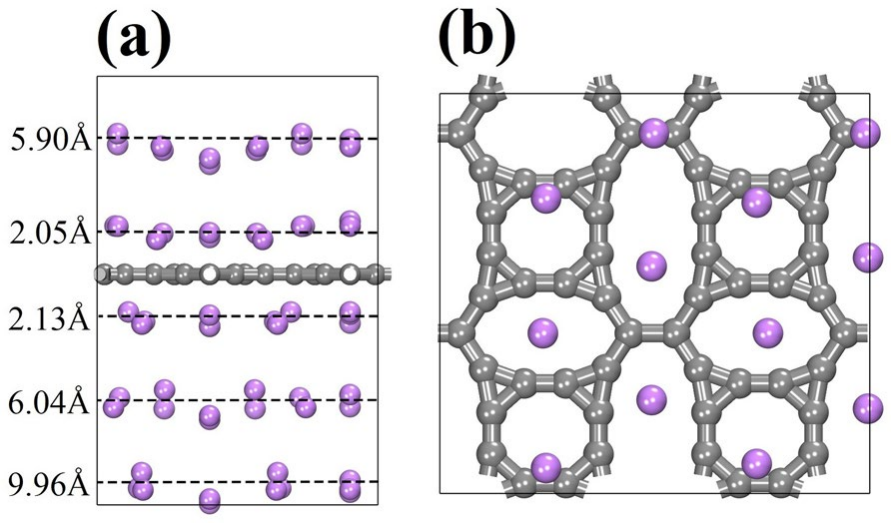
(**a**) Side view for the adsorption of multiple Li atoms on TODD-G. Panel (**b**) illustrates the top view for the first Li atom layer adsorbed at 2.05 Å from the material plane. This figure was prepared using the Materials Studio Visualizer^51^.

Figure 13a reveals that multiple Li atoms can be effectively adsorbed on the TODD-G surface, forming distinct layers upon reaching their total adsorption capacity at a specific distance from the material plane. The first layer of Li atoms adsorbed above/below the material plane at 2.05/2.13 Å tends to localize each Li atom at the center of the rings, as depicted in Fig. 13b.

## Methods

The CASTEP code^78^, implemented in Biovia Materials Studio software^51^, was used to facilitate the execution of DFT and AIMD simulations, with a focus on investigating the thermomechanical and optoelectronic properties of TODD-G. The generalized gradient approximation (GGA) was employed to address exchange-correlation functionals. Specifically, we applied the Perdew-Burke-Ernzerhof (PBE)^79^ and hybrid Heyd–Scuseria–Ernzerhof (HSE06)^80^ functionals. To address interactions among nuclear electrons, norm-conserving pseudopotentials within CASTEP were employed.

A 600 eV energy cutoff and a 1.0 $\times$ 10^-5^ eV convergence criterion were employed for electronic self-consistency. During TODD-G lattice relaxation, the force on each atom was maintained below 1.0 $\times$ 10^-3^ eV/Å. The optimization procedure considered a fixed base vector along the z-direction with a $10\times 10\times 1$ k-point grid. Electronic and optical calculations employed a $15\times 15\times 1$ k-point grid for GGA/PBE and $5\times 5\times 1$ for HSE06. PDOS calculations at the HSE06 level utilized a $20\times 20\times 1$ k-point grid. Elastic properties were determined using the LDA/CA-PZ method^81,82^. A 20 Å vacuum region prevented unwanted interactions between periodic images.

The phonon characteristics were analyzed using a linear response method with a grid spacing of 0.05 Å, ensuring convergence with a 10^-5^ eV/Å^2^ of tolerance. Mechanical properties were analyzed using a stress-strain approach based on the Voigt-Reuss-Hill method^83,84^. Stability tests using AIMD simulations used a $2\times 2\times 1$ supercell with 56 atoms and a fixed time step of 1.0 fs over 5.0 ps. Temperature control was employed with the Nosé-Hoover thermostat^85^, consistent with other AIMD studies^86,87^. In the TODD-G/Li cases, we have incorporated vdW corrections within the Grimme scheme^88^.

To explore TODD-G’s optical characteristics, an external electric field (1.0 V/Å) was applied in the x, y, and z directions. The optical properties were derived using the complex dielectric constant $\epsilon =\epsilon _1+i\epsilon _2$ with $\epsilon _1$ and $\epsilon _2$ representing the real and imaginary components. This analysis followed the methodology outlined in reference^52^.

Fully atomistic reactive MD simulations were conducted using the AIREBO^89^ potential, as implemented in LAMMPS^90,91^, to determine the melting point of TODD-G. This potential is widely recognized for its effectiveness in modeling carbon-based nanostructure’s mechanical properties and thermal stability once it can handle reconstructions and ruptures at the atomic level of bonds. The MD snapshots were rendered using VMD software^39^.

The thermal stability investigation of TODD-G involved a gradual heating process from 300 to 5000 K, conducted through heating ramp simulations. In the initial simulation phases, the energy of TODD-G was minimized. Keeping zero pressure and 300 K, we performed integration within the constant NPT ensemble for 50 ps to eliminate any residual stress. Subsequently, all systems were subjected to an NVT ensemble for 100 ps. These simulations were executed with a time step set at 0.05 fs.

## Conslusions

Through DFT, AIMD, and classical reactive MD simulations, we introduce TODD-G, a novel 2D flat carbon material with a porous topology comprising 3-8-10-12 carbon rings. This structure demonstrates programmable metallic properties designed using a bottom-up approach. TODD-G exhibits a low-energy structure that ensures dynamic, thermal, and mechanical stability. It has an average diffusion barrier (about 0.85 eV) and a metallic framework boasting excellent conductivity. This trend suggests that TODD-G can be designed for superior lithium-ion battery efficiency.

A Dirac cone appears above the Fermi level in the TODD-G band structure, and the DOS near the Fermi level primarily originates from 2pz atomic orbitals, confirming its non-magnetic metallic nature. TODD-G exhibits noticeable in-plane anisotropy in electronic, mechanical, and optical properties, aligning with its topology. Its inherent porosity is attributed to the presence of 8-10-12-atom rings and the rigidity of bonds within fused trigonal rings, contributing to its reduced mechanical resilience compared to graphene. Results from classical reactive (ReaxFF) molecular dynamics simulations at 1800 K indicate structural integrity with no bond reconstructions.

With its metallic signature and electronic states characterized by remarkable delocalization, TODD-G holds promise for applications in nanoelectronics and photonics. The favorable thermal stability and diminished lattice thermal conductivity make it a potential solution for overcoming thermal management issues in microelectronics. The unique anisotropic mechanical properties of TODD-G offer opportunities to bolster the strength of lightweight materials. Furthermore, its noteworthy UV absorption combined with low reflectivity establishes TODD-G as a compelling choice for effective UV collection, especially in applications related to sensing and detection.

### Supplementary Information

Supplementary Information.

Supplementary Video 1.

Supplementary Video 2.

Supplementary Video 3.

## Data Availability

Data supporting this study’s findings are available upon reasonable request from the last author L.A.R.J.
