# Supplementary material for: Proposing TODD-graphene as a novel porous 2D carbon allotrope designed for superior lithium-ion battery efficiency
Source: Sci Rep. 2024 Mar 14;14:6202. doi: 10.1038/s41598-024-56312-x (PMC10940596; doi:10.1038/s41598-024-56312-x)
Supplement: Supplementary file 1 — Supplementary Information. [file 41598_2024_56312_MOESM1_ESM.pdf]

Supplementary Information for
TODD-Graphene: A Novel Porous 2D Carbon Allotrope for High-Performance Lithium-Ion Batteries

E. J. A. dos Santos,[1,2] K. A. Lopes Lima,[1,2] and L. A. Ribeiro Junior[1,2]

[1]*Institute of Physics, University of Brasília, 70910-900, Brasília, Brazil*

[2]*Computational Materials Laboratory, LCCMat, Institute of Physics, University of Brasília, 70910-900, Brasília, Brazil*

I. ELECTRON AND HOLE MOBILITY IN TODD-G

To gain a thorough insight into the electronic conductance in TODD-G, we have expanded our examination to consider charge carrier mobility in this material. The analysis uses the deformation potential (DP) theory [1], a well-established approach based on the effective mass approximation []. This method is widely employed for assessing carrier mobility in 2D layered semiconductors. In this way, the carrier mobility (μ) is defined as follows:

$$\mu = \frac{e\hbar C_{2D}}{k_B T m_i^* m_d \left(E_1\right)^2}. \tag{1}$$

In the expression for the average effective mass, $m_d = \sqrt{m_x^* m_y^*}$. Here, C_{2D} denotes the in-plane stiffness, defined as $C_{2D} = \left(\partial^2 E/\partial \delta^2\right)/S_0$, where E, δ, and S_0 represent the total energy, applied strain, and the area of the system, respectively.

The effective masses for electrons (e) and holes (h) are denoted by m_i^*. This parameter is calculated by fitting the band dispersion to

$$m^* = \hbar^2 \left(\frac{\partial^2 E(k)}{\partial k^2}\right)^{-1}. \tag{2}$$

Additionally, E_1 is calculated as $dE_{edge}/d\delta$, where δ represents the applied strain in increments of 0.5%. The term E_{edge} corresponds to the energy of the band edges, specifically the valence band maximum (VBM) for holes and the conduction band minimum (CBM) for electrons. The positions of VBM and CBM are identified at the G and Z points. Furthermore, in the context of the equation, k_B, T, e, and $\hbar$ denote Boltzmann's constant, temperature, the elementary charge of an electron, and Planck's constant, respectively.

We have consolidated the computed values for m_i^*, m_d, C_{2D}, E_1, and μ at 300 K, presenting them

in Table S1. The determination of E_1 involves subjecting the system to both compressive and tensile strains, followed by a linear fitting of CBM and VBM values for electrons and holes, respectively (refer to Figure S1(a)). Subsequently, C_{2D} is derived using a quadratic fitting approach applied to the total energy data in response to compressive and tensile strains, as illustrated in Figure S1(b).

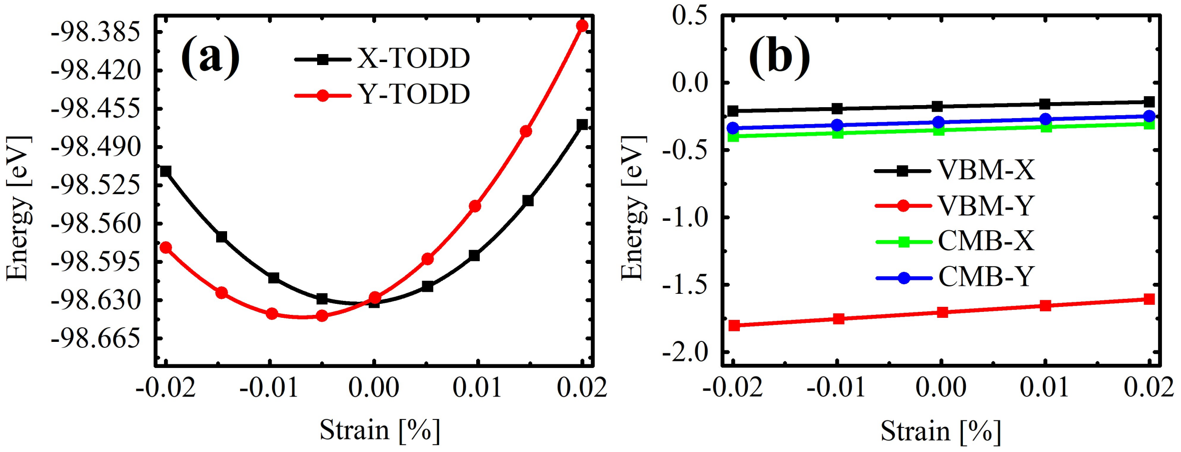

FIG. S1. Panel (a) depicts the quadratic fitting curve for the TODD-G total energy concerning the applied compressive and tensile strains, enabling the derivation of the in-plane stiffness (C_{2D}). Meanwhile, Figure (b) presents the linear fitting diagram for the TODD-G total energy concerning the compressive and tensile strains, facilitating the determination of the deformation potential constant (E_1).

In TODD-G, the effective masses for holes (m_h^*) and electrons (m_e^*) are $1.04m_0$ and $0.46m_0$ in the x-direction and $1.13m_0$ and $0.49m_0$ in the y-direction, respectively, with m_0 denoting the electron mass. It is worth mentioning that the m_h^* and m_e^* values observed in TODD-G are similar to those reported for pristine and bilayer graphene (about 0.012-0.43m_0 for electrons) [2–4].

TABLE S1. Calculated effective mass m_i^*, average effective mass m_d, in-plane stiffness C_{2D}, DP constant E_1, and charge carrier mobility m_i for TODD-G. Here, m_0 is the effective mass of a free electron.

System	Carrier	m^* (m_0)	m_d (m_0)	C_{2D} (eV/Å)	$E1$ (eV)	μ (10^3 cm^2 V^{-1} s^{-1})
TODD	e	0.46	0.37	7.67	2.29	89.25
x-direction	h	1.04	0.47	7.67	1.70	11.13
TODD	e	0.49	0.37	7.87	2.21	77.23
y-direction	h	1.13	0.47	7.87	4.92	10.16

The electron mobility outperforms the hole mobility, as highlighted in Table S1. The carrier mobility within the TODD-G monolayer displays anisotropic behavior, showcasing differences between the x and y directions. In the case of TODD-G, the carrier mobility along the x direction exceeds the one along the y direction. This distinction primarily arises from the smaller effective mass of carriers in

the x direction compared to the y direction.

---

[1] J Bardeen and WJPR Shockley. Deformation potentials and mobilities in non-polar crystals. *Physical review*, 80(1):72, 1950.

[2] E Tiras, S Ardali, T Tiras, E Arslan, S Cakmakyapan, O Kazar, Jawad Hassan, Erik JanzÈn, and E Ozbay. Effective mass of electron in monolayer graphene: Electron-phonon interaction. *Journal of Applied Physics*, 113(4), 2013.

[3] K. Zou, X. Hong, and J. Zhu. Effective mass of electrons and holes in bilayer graphene: Electron-hole asymmetry and electron-electron interaction. *Phys. Rev. B*, 84:085408, Aug 2011.

[4] AZ AlZahrani and GP Srivastava. Graphene to graphite: electronic changes within dft calculations. *Brazilian Journal of Physics*, 39:694–698, 2009.
